# Residence-based inequalities in overweight/obesity in sub-Saharan Africa: a multivariate non-linear decomposition analysis

**DOI:** 10.1186/s41182-024-00593-5

**Published:** 2024-04-07

**Authors:** Priscilla Atsu, Aliu Mohammed, Collins Adu, Richard Gyan Aboagye, Bright Opoku Ahinkorah, Abdul-Aziz Seidu

**Affiliations:** 1 Department of Health Promotion, Education and Disability Studies, School of Public Health, Kwame Nkrumah University of Science and Technology, Kumasi, Ghana; 2 Department of Health, Physical Education and Recreation, University of Cape Coast, Cape Coast, Ghana; 3 Centre for Social Research in Health, University of New South Wales, Sydney, NSW Australia; 4 School of Population Health, University of New South Wales, Sydney, NSW 2052 Australia; 5 Department of Family and Community Health, Fred N. Binka School of Public Health, University of Health and Allied Sciences, Hohoe, Ghana; 6 School of Clinical Medicine, University of New South Wales Sydney, Sydney, Australia; 7 School of Public Health, Faculty of Health, University of Technology Sydney, Sydney, Australia; 8 REMS Consultancy Services, Sekondi-Takoradi, Western Region Ghana; 9 Centre for Gender and Advocacy, Takoradi Technical University, Takoradi, Ghana; 10 College of Public Health, Medical and Veterinary Sciences, James Cook University, Townsville, QLD 4811 Australia

**Keywords:** Body mass index, Global health, Sub-Saharan Africa, Women, Demographic and Health Survey

## Abstract

**Background:**

Overweight/obesity remains a major risk factor for non-communicable diseases and their associated morbidities and mortalities. Yet, limited studies have comprehensively examined factors contributing to the rural–urban disparities in overweight/obesity among women in sub-Saharan Africa. Thus, our study sought to decompose the rural–urban disparities in overweight/obesity among women in sub-Saharan Africa (SSA) using nationally representative datasets.

**Methods:**

We performed a cross-sectional analysis of data from the Demographic and Health Surveys of 23 sub-Saharan African countries conducted from 2015 to 2022. A sample of 177,329 women was included in the analysis. Percentages with confidence intervals (CIs) were used to summarize the prevalence of overweight/obesity per rural–urban strata and pooled level. A multivariate non-linear decomposition analysis was used to identify the factors contributing to the rural–urban disparities in overweight/obesity. The results were presented using coefficients and percentages.

**Results:**

The pooled prevalence of overweight/obesity among the women was higher in urban areas (38.9%; 95% CI = 38.2–39.6) than rural areas (19.1%; 95% CI = 18.7–19.6). This pattern was observed in all the countries surveyed, except in South Africa, where women in rural areas (53.1%; 95% CI = 50.0–56.4) had a higher prevalence of overweight/obesity than those in urban areas (46.0%; 95% CI = 43.2–48.9). Approximately 54% of the rural–urban disparities in overweight/obesity was attributable to the differences in the women’s characteristics or explanatory variables. More than half of the rural–urban disparities in overweight/obesity would be reduced if the disparities in women’s characteristics were levelled. Among the women’s characteristics, frequency of watching television (29.03%), wealth index (26.59%), and level of education (9.40%) explained approximately 65% of the rural–urban differences in overweight/obesity.

**Conclusion:**

The prevalence of overweight/obesity among women in SSA remains high and skewed towards women in urban areas. Increased frequency of watching television, high wealth index, and higher educational attainment contributed largely to the rural-urban disparities in overweight/obesity among women in SSA. Thus, interventions aimed at reducing overweight/obesity among women in SSA could be targeted at reducing the frequency of television watching as well as promoting physical activities among wealthy women and those with higher education, particularly in urban areas.

**Supplementary Information:**

The online version contains supplementary material available at 10.1186/s41182-024-00593-5.

## Introduction

Globally, overweight/obesity remains a major risk factor for non-communicable diseases (NCDs) and their associated morbidities and mortalities [1]. NCDs are the leading causes of death worldwide, accounting for about 41 million people (71% of total global deaths) annually [2]. The rapid increase in the prevalence of NCDs remains a major public health concern in most low- and middle-income countries (LMICs), especially those in sub-Saharan Africa (SSA) [3]. Between 1990 and 2017, the contribution of NCDs to the total burden of diseases in SSA increased from 18.6 to 29.8%, while the total disability-adjusted life years attributable to NCDs also increased by 67% (from 90.6 to 151.3 million) [4]. Thus, despite being largely preventable through lifestyle modifications and the promotion of healthy diets [5], overweight/obesity contributes significantly to the burden of diseases in SSA [4].

Overweight/obesity, described as an abnormal build-up of fat in the body that can affect health [1], occurs due to energy disequilibrium between calories consumed and calories expended [6]. The body mass index (BMI) is used to determine and classify overweight/obesity among adults. Adults with BMI of ≥ 25 kg/m^2^ are classified as overweight, while those with a BMI of ≥ 30 kg/m^2^ are obese [7]. Overweight and obesity constitute major risk factors for several NCDs, including diabetes mellitus, cardiovascular diseases, kidney diseases, and some cancers [8]. Most deaths associated with cardiovascular diseases are attributable to being overweight/obese [9].

The global prevalence of overweight/obesity continues to increase persistently despite the numerous interventions in many countries aimed at dealing with the phenomenon [7, 10, 11]. As of 2022, 1 in 8 people in the world were living with obesity [5]. Also, 2.5 billion and 890 million adults aged 18 years and older were overweight and obese, respectively [5]. It is estimated that if the current trend continues, approximately 57.8% of the global adult population could be overweight/obese by the year 2030 [12]. This could increase the burden of NCDs to alarming proportions and thus, compound the burden of diseases in SSA, where communicable diseases such as malaria, tuberculosis, and HIV/AIDS remain high [4].

Though influenced by genetic predisposition, overweight/obesity is triggered by human behaviours such as poor diet and physical inactivity or sedentary lifestyle and urbanization [6, 7]. Several studies have been conducted to assess the predictors of overweight/obesity among women [13–24]. These previous studies identified various factors, including age, place of residence, educational status, wealth status, marital status, and watching television, to be associated with overweight/obesity among women. However, an understanding of the residence-based inequalities in overweight/obesity among women has received little attention, especially in the context of SSA. With place of residence, most of the findings showed a higher likelihood of overweight/obesity among urban women compared to women who lived in rural areas [15, 24]. However, the extent of this disparity is unknown. Not knowing the extent of residence-based inequality along with its underlying factors can misguide policy priorities. Consequently, it is crucial to assess the extent of residence-based inequalities in overweight/obesity and determine the factors that contribute to the observed disparities among women in SSA. The study’s findings could help policymakers design and prioritize various interventions, programmes, and strategies to address the issue of overweight/obesity among women in SSA.

## Materials and methods

### Data source and study design

We performed secondary data analyses from the most recent Demographic and Health Surveys (DHS) of 23 countries in SSA. Countries whose most recent DHS was conducted from 2015 to 2022 and had data on all variables of interest were included in the study. The DHS is a nationally representative survey conducted in over 90 LMICs across the globe [25]. It utilizes cross-sectional study design and the respondents: women, men, and children were sampled using a two-stage cluster sampling method. The detailed description of the DHS methodology, including the design, sampling, and data collection techniques have been highlighted in the literature [26, 27]. To gather information from the respondents on a variety of health and demographic factors, including overweight/obesity, pretested and structured questionnaires were used [26, 27]. In designing this study, we referred to the Strengthening Reporting of Observational Studies in Epidemiology guidelines [28]. The dataset used for this study can be accessed after registration at https://dhsprogram.com/data/available-datasets.cfm [29]. Overall, 177,329 women aged 15–49 years were included in the study (Table 1).

**Table 1:** Sample distribution per country

Country		Survey year	Weighted sample (*n*)	Weighted percentage (%)
1.	Burkina Faso	2021	8283	4.67
2.	Benin	2017–18	7447	4.20
3.	Burundi	2016–17	8052	4.54
4.	Cote d’Ivoire	2021	6973	3.93
5.	Cameroon	2018	6862	3.87
6.	Ethiopia	2016	7293	4.11
7.	Gabon	2019–21	5339	3.01
8.	Gambia	2019–20	5633	3.18
9.	Guinea	2018	5065	2.86
10.	Kenya	2019–21	14,964	8.44
11.	Liberia	2019–20	3866	2.18
12.	Madagascar	2021	8829	4.98
13.	Mali	2018	4844	2.73
14.	Mauritania	2019–2021	7319	4.13
15.	Malawi	2015–16	11,419	6.44
16.	Nigeria	2018	19,289	10.88
17.	Rwanda	2019–20	6757	3.81
18.	Sierra Leone	2019	7292	4.11
19.	Chad	2014–15	8345	4.71
20.	Tanzania	2015–16	6189	3.49
21.	Uganda	2016	8655	4.88
22.	South Africa	2018	3976	2.24
23.	Zimbabwe	2015	4638	2.62
	All countries	2015–2022	177,329	100.00

### Study variables

#### Outcome variable

Overweight/obesity was the outcome variable for this study. It was calculated by dividing the weight by the height squared of each respondent and the result was expressed as kilogrammes/meter^2^ (kg/m^2^). Following the World Health Organization’s [5] standard for BMI cut-off points: underweight, < 18.5 kg/m^2^; normal weight, 18.5–25 kg/m^2^; overweight, 25.0–29.9 kg/m^2^; and obese, ≥ 30.0 kg/m^2^, we categorized those whose BMI was ≥ 25.0 kg/m^2^ as being overweight/obese and coded it as “1 = yes” and those whose BMI was < 25.0 kg/m^2^ as not overweight/obesity and was coded as “0 = no”. This categorisation is consistent with that of previous studies that utilized the DHS dataset in SSA [19, 22, 23].

#### Inequality stratifier

Place of residence was used as the inequality stratifier. In the DHS, the respondents were asked to indicate their type of residence and the response options were urban and rural. We recoded the response options into 0 = rural and 1 = urban in the final analysis.

#### Explanatory variables

Eight explanatory variables were considered in this study. These variables were selected based on their significant associations with overweight/obesity from literature [15–24] as well as their availability in the DHS dataset. The variables consisted of the age of the women, level of education, marital status, current working status, parity, frequency of watching television, household size, and wealth index. The detailed categories of the variables can be found in Table 2.

**Table 2:** Distribution of sample across the explanatory variables

Variables	Pooled weighted *Sample* (Percentage)	Rural weighted *Sample* (Percentage)	Urban weighted *Sample* (Percentage)
Women’s age (years)			
15–19	39,276 (22.1)	24,243 (22.3)	15,044 (21.9)
20–24	30,014 (16.9)	17,503 (16.1)	12,466 (18.1)
25–29	28,329 (16.0)	16,719 (15.4)	11,577 (16.8)
30–34	24,623 (13.9)	14,891 (13.7)	9723 (14.1)
35–39	22,477 (12.7)	13,811 (12.7)	8669 (12.6)
40–44	17,654 (10.0)	11,324 (10.5)	6358 (9.2)
45–49	14,956 (8.4)	10,022 (9.3)	4980 (7.3)
Level of education			
No education	52,691 (29.7)	41,069 (37.9)	12,086 (17.6)
Primary	54,057 (30.5)	39,973 (36.8)	14,448 (21.0)
Secondary	58,852 (33.2)	24,932 (23.0)	33,337 (48.4)
Higher	11,729 (6.6)	2538 (2.3)	8946 (13.0)
Marital status			
Never in union	54,873 (30.9)	27,871 (25.7)	26,702 (38.8)
Married	87,971 (49.6)	59,811 (55.1)	28,475 (41.4)
Living with partner	17,837 (10.1)	10,919 (10.1)	6918 (10.1)
Widowed	4820 (2.7)	3191 (2.9)	1641 (2.4)
Divorced	4297 (2.4)	2393 (2.2)	1892 (2.7)
Separated	7531 (4.3)	4327 (4.0)	3189 (4.6)
Current working status			
Not working	72,370 (40.8)	41,502 (38.2)	30,722 (44.6)
Working	104,959 (59.2)	67,010 (61.8)	38,095 (55.4)
Parity			
Zero birth	51,659 (29.1)	27,468 (25.3)	23,972 (34.8)
One birth	23,968 (13.5)	13,058 (12.0)	10,826 (15.7)
Two births	22,722 (12.8)	12,860 (11.9)	9807 (14.3)
Three births	20,383 (11.5)	12,317 (11.4)	8058 (11.7)
Four or more births	58,597 (33.1)	42,809 (39.4)	16,154 (23.5)
Frequency of watching television			
Not at all	94,435 (53.2)	78,333 (72.2)	17,184 (25.0)
Less than once a week	26,565 (15.0)	14,261 (13.1)	12,199 (17.7)
At least once a week	56,329 (31.8)	15,918 (14.7)	39,435 (57.3)
Household size			
Small	81,790 (46.1)	48,192 (44.4)	33,501 (48.7)
Medium	74,644 (42.1)	47,951 (44.2)	26,812 (39.0)
Large	20,895 (11.8)	12,369 (11.4)	8504 (12.3)
Wealth index			
Poorest	29,547 (16.7)	27,519 (25.4)	2524 (3.7)
Poorer	32,011 (18.1)	28,334 (26.1)	4137 (6.0)
Middle	34,477 (19.4)	26,274 (24.2)	8476 (12.3)
Richer	37,759 (21.3)	18,950 (17.5)	18,590 (27.0)
Richest	43,535 (24.5)	7435 (6.8)	35,089 (51.0)

### Statistical analyses

Stata software version 17.0 (Stata Corporation, College Station, TX, USA) was used to perform all the statistical analyses. We used percentages to summarize the prevalence of overweight/obesity among the women in SSA and the results were presented using spatial maps. We used cross-tabulation to examine the distribution of overweight/obesity across the explanatory variables. Pearson chi-square test of independence was used to identify the variables that were significantly associated with overweight/obesity. We used a four-modelled multilevel binary logistic regression analysis to examine the factors associated with overweight/obesity among women in SSA. Model I had no explanatory variables as it depicts the variation in overweight/obesity attributable to the clustering at the primary sampling unit (PSU). Model II and III contained the individual (age of the women, level of education, marital status, current working status, parity, and frequency of watching television) and contextual level (household size, wealth index, and place of residence) variables, respectively. Model IV contained all the explanatory variables. Fixed and random effects results were generated. The fixed effect results showed the association between the explanatory variables and overweight/obesity, whereas the random effect indicated the fitness of the four models. Adjusted odds ratio (aOR) with their respective 95% confidence intervals (CI) were used to present the fixed effect results. For model fitness, we used the Akaike Information Criterion (AIC) and log-likelihood values. The model with the least AIC and highest log-likelihood values was selected as the best-fitted model. Next, we used a multivariable binary logistic regression to examine the factors associated with overweight/obesity among the women per the rural–urban strata. The results were presented as aOR with their respective 95% CIs. We also weighted all the analyses and Stata’s “svyset” command was used to adjust for disproportionate sampling and survey design.

### Decomposition analysis

A multivariate non-linear decomposition analysis was used to identify the factors contributing to the rural–urban disparities in overweight/obesity [30]. Evidence has shown that decomposition analysis is often used to measure the contributions to group differences in the average predictions from multivariate models [30]. Powers et al. [30] posit that a multivariate decomposition analytical method splits the components of a group difference in a statistic, such as means or proportions, into two categories: one for compositional differences between groups, or differences in characteristics, and another for differences in the effects of characteristics, or differences in coefficient. This method was used instead of the well-known Blinder–Oaxaca decomposition which typically assumes linear relationships, applied to linear models and often used for wage gap analysis.

The equation for the decomposition can be expressed as follows:

$$Y=\alpha +{\sum }_{i=1}^{k}{\beta }_{i}{X}_{i}+\epsilon ,$$

where $Y$ is the outcome variable which is overweight/obesity; $\alpha$ is the intercept; ${\beta }_{i}$ is the coefficient vector associated with each of the observed characteristics. ${X}_{i}$ is the vector of observed characteristics (women’s age, marital status, level of education, current working status, parity, frequency of watching television, household size, and wealth index); $\epsilon$ is the error term.

The rural–urban differences in the overweight/obesity ($D$) can be expressed as:

$$D={Y}_{{\text{urban}}}-{Y}_{{\text{rural}}}.$$

The difference as a result of the decomposition results in the contributions of each observed characteristic ($${X}_{i}):$$

$$D={\sum }_{i=1}^{k}{\beta }_{i}\left({{X}_{i}}_{{\text{urban}}}-{{X}_{i}}_{{\text{rural}}}\right)+ {\sum }_{i=1}^{k}{\beta }_{i}\left({\overline{\overline{{X}_{i}}}}_{{\text{urban}}}-{\overline{\overline{{X}_{i}}}}_{{\text{rural}}}\right)+ \left({\epsilon }_{{\text{urban}}}-{\epsilon }_{{\text{rural}}}\right),$$

where ${{X}_{i}}_{{\text{urban}}}\; {\text{and}}\; {{X}_{i}}_{{\text{rural}}}$ are the means of each characteristic in the urban and rural groups; ${\overline{\overline{{X}_{i}}}}_{{\text{urban}}}\; {\text{and}} \;{\overline{\overline{{X}_{i}}}}_{{\text{rural}}}$ are the coefficients associated with each observed characteristic; ${\epsilon }_{{\text{urban}}} \; {\text{and}}\; {\epsilon }_{{\text{rural}}}$ are the error terms for urban and rural groups.

### Ethical consideration

We used secondary data for this study; hence, ethical clearance was not sought for our study since the datasets are freely available in the public domain. Prior to this study, we sought permission from the Monitoring and Evaluation to Assess and Use Results Demographic and Health Surveys (MEASURE DHS) to access and use the datasets and it was granted.

## Results

### Prevalence of overweight/obesity among the women in sub-Saharan Africa

**Fig. 1:**
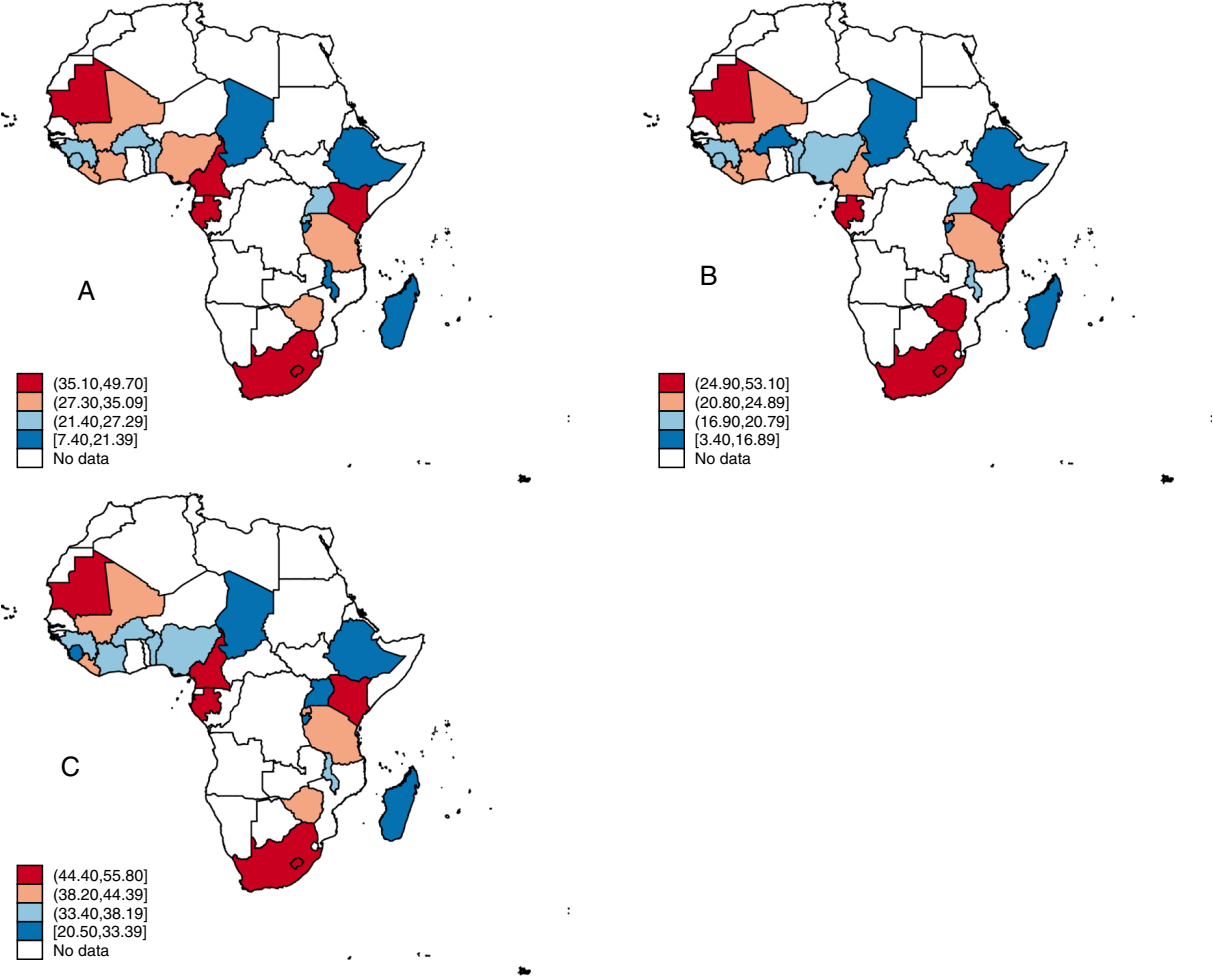
Prevalence of overweight/obesity in sub-Saharan Africa (**A**); rural sub-Saharan Africa (**B**); and urban sub-Saharan Africa (**C**)

Figure 1 and Additional file 1: Table S1 show the prevalence of overweight/obesity among women in 23 countries in SSA and across the explanatory variables. The findings revealed that the pooled prevalence of overweight/obesity among the women surveyed across the 23 countries was 27.0% (95% CI = 26.6–27.5). For the rural–urban disparities, the pooled prevalence of overweight/obesity among the women was higher in urban areas (38.9%; 95% CI = 38.2–39.6) than in rural areas (19.1%; 95% CI = 18.7–19.6) (Additional file 1: Table S1). The countries with the highest proportions of overweight/obesity were Cameroon, Gambia, Kenya, Mauritania, and South Africa. For rural residents, the proportions of overweight/obesity were highest in Gabon, Kenya, Liberia, Mauritania, South Africa, and Zimbabwe. Cameroon, Gabon, Kenya, Mauritania, and South Africa had the highest proportions of overweight/obesity in urban SSA (Fig. 1). We observed statistically significant differences in overweight/obesity across all the explanatory variables, for the pooled sample. Similar patterns were observed across the rural and urban settings (Additional file 1: Table S1).

### Distribution of sample across the explanatory variables

Table 2 presents the distribution of the sample across the explanatory variables. The modal age of the women was 15–19 years (22.1%), almost half were married (49.6%), and the majority were working (59.2%). Also, the modal parity was four or more births (33.1%), majority of the women did not watch television at all (53.2%), and the modal wealth index was the richest wealth index (24.5%). For the distribution of overweight/obesity across the explanatory variables (Additional file 1: Table S1), we observed that the prevalence of overweight/obesity was higher among women aged 45–49 years (39.5%), those with higher education (45.5%), those who were divorced (34.3%), those who were working (29.3%), those with three births (35.5%), those who watched television at least once a week (41.1%), those who lived in small households (29%), and those who belonged to the richest wealth index (41.2%).

### Factors associated with overweight/obesity among women in sub-Saharan Africa

Table 3 shows the results of the factors associated with overweight/obesity among women in SSA. The results here are presented from Model IV, which was the best fit model. The odds of overweight/obesity increased with age, with the highest odds among women aged 45–49 (aOR = 6.89, 95% CI = 6.28–7.55). Married (aOR = 1.21, 95% CI = 1.14–1.30), cohabiting (aOR = 1.19, 95% CI = 1.10–1.28), and divorced (aOR = 1.24, 95% CI = 1.11–1.38) women had higher odds of overweight/obesity compared to never in union women. The odds of overweight/obesity was highest among women who had attained secondary level of education (aOR = 1.68, 95% CI = 1.53–1.79) compared to those with no education. Women with one or more births had higher odds for overweight/obesity compared to those with zero birth, with the highest odds among women with three births (aOR = 1.30, 95% CI = 1.20–1.41). Additionally, women who watched television less than once a week (aOR = 1.51, 95% CI = 1.44–1.58) and at least once a week (aOR = 1.97, 95% CI = 1.88–2.06) were more likely to be overweight/obese compared to those who never watched television. The odds of overweight/obesity also increased with wealth index, with the highest odds among women living in richest households (aOR = 2.01, 95% CI = 1.87–2.15). Women living in urban areas reported higher odds for overweight/obesity compared to those in rural areas (aOR = 1.64, 95% CI = 1.56–1.73). However, lower odds of overweight/obesity was found among women who were working compared to those who were not working (aOR = 0.93, 95% CI = 0.89–0.96).

**Table 3:** Factors associated with overweight/obesity among women in sub-Saharan Africa

Variable	Model I Empty model	Model II aOR [95% CI]	Model III aOR [95%CI]	Model IV aOR [95%CI]
Fixed effect results				
Women’ s age (years)				
15–19		1.00		1.00
20–24		1.81*** [1.69, 1.94]		1.75*** [1.63, 1.87]
25–29		3.21*** [2.98, 3.46]		2.96*** [2.75, 3.20]
30–34		5.04*** [4.65, 5.47]		4.51*** [4.15, 4.89]
35–39		6.56*** [6.03, 7.14]		5.74*** [5.27, 6.25]
40–44		7.12*** [6.53, 7.78]		6.25*** [5.72, 6.83]
45–49		7.89*** [7.21, 8.63]		6.89*** [6.28, 7.55]
Marital status				
Never in union		1.00		1.00
Married		1.20*** [1.13, 1.28]		1.21*** [1.14, 1.30]
Cohabiting		1.16*** [1.07, 1.26]		1.19*** 1.10, 1.28]
Widowed		1.02 [0.92, 1.13]		1.02 [0.92, 1.14]
Divorced		1.27*** [1.14, 1.41]		1.24*** [1.11, 1.38]
Separated		1.04 [0.95, 1.15]		1.05 [0.96, 1.16]
Level of education				
No education		1.00		1.00
Primary		1.48*** [1.42, 1.55]		1.41***[1.35, 1.48]
Secondary		2.03*** [1.93, 2.13]		1.68*** [1.60, 1.76]
Higher		2.15*** [1.99, 2.33]		1.65***[1.53, 1.79]
Current working status				
Not working		1.00		1.00
Working		0.92*** [0.89, 0.95]		0.93*** [0.89, 0.96]
Parity				
None		1.00		1.00
One birth		1.14*** [1.06, 1.22]		1.18*** [1.10, 1.27]
Two births		1.19*** [1.11, 1.29]		1.27*** [1.18, 1.38]
Three births		1.19*** [1.10, 1.29]		1.30*** [1.20, 1.41]
Four or more births		1.02 [0.95, 1.10]		1.18*** [1.09, 1.28]
Frequency of watching television				
Not at all		1.00		1.00
Less than once a week		1.87*** [1.79, 1.97]		1.51*** [1.44, 1.58]
At least once a week		2.84*** [2.72, 2.97]		1.97*** [1.88, 2.06]
Household size				
Small			1.00	1.00
Medium			0.91*** [0.88, 0.94]	0.98 [0.94, 1.02]
Large			0.83*** [0.79, 0.88]	0.97 [0.91, 1.03]
Wealth index				
Poorest			1.00	1.00
Poorer			1.37*** [1.29, 1.46]	1.31*** [1.23, 1.39]
Middle			1.74*** [1.64, 1.85]	1.54*** [1.45, 1.64]
Richer			2.22*** [2.09, 2.37]	1.87*** [1.75, 2.00]
Richest			2.68*** [2.51, 2.87]	2.01*** [1.87, 2.15]
Type of place of residence				
Rural			1.00	1.00
Urban			1.90*** [1.81, 1.99]	1.64*** [1.56, 1.73]
Random effect model				
PSU variance (95% CI)	0.347 [0.310, 0.388]	0.209 [0.181, 0.243]	0.253 [0.223, 0.287]	0.214 [0.185, 0.247]
ICC	0.095	0.060	0.071	0.061
Wald Chi-square	Reference	8695.52	3003.78	9074.98
Model fitness				
Log-likelihood	− 217,118.92	− 191,804.6	− 207,041.47	− 188,612.12
AIC	434,241.8	383,655.2	414,191.9	377,284.2
*N*	177,329	177,329	177,329	177,329
Number of clusters	1691	1691	1691	1691

aOR: adjusted odds ratios; CI: confidence interval; 1.00: reference category; PSU: primary sampling unit; ICC: intra-class correlation coefficient; AIC: Akaike Information Criterion

*p < 0.05, **p < 0.01, ***p < 0.001

### Factors associated with overweight/obesity among women in rural and urban sub-Saharan Africa

**Fig. 2:**
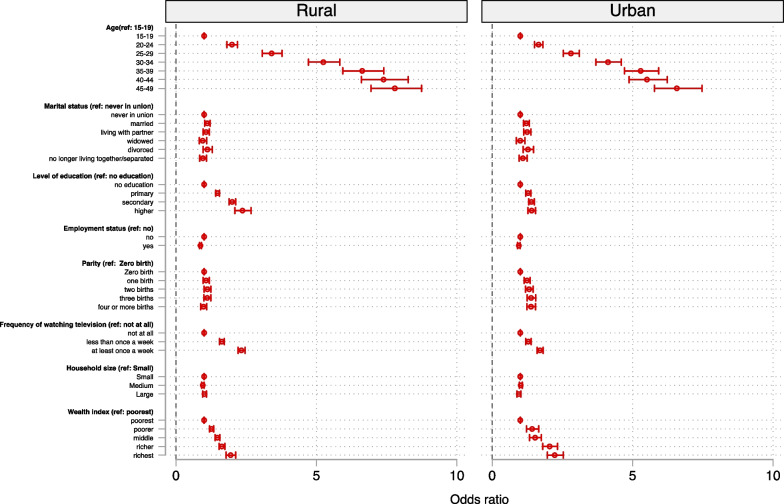
Predictors of overweight/obesity among women in rural and urban sub-Saharan Africa

In Fig. 2, we present the results of the factors associated with overweight/obesity among women in rural and urban areas. We found that the odds of overweight/obesity increased with advancing age, with the highest likelihood among women aged 45–49 in both rural (aOR = 7.79, 95% CI = 6.94–8.74) and urban (aOR = 6.57, 95% CI = 5.78–7.47) areas. Also, the odds of overweight/obesity increased with increasing level of education in both rural (aOR = 2.36, 95% CI = 2.09–2.67) and urban areas (aOR = 1.40, 95% CI = 1.28–1.54), with those with higher educational level recording the highest odds. However, working women were less likely to be overweight/obese in rural (aOR = 0.87; 95% CI = 0.83–0.91) and urban (aOR = 0.95, 95% CI = 0.90–0.99) areas. With marital status, the odds of overweight/obesity were higher among those divorced (aOR = 1.27, 95% CI = 1.10–1.47), those married (aOR = 1.21, 95% CI = 1.12-1.32), and those living with their partners (cohabiting) (aOR = 1.24, 95% CI = 1.12–1.38) in urban areas only whereas married women in rural areas (aOR = 1.11, 95% CI = 1.02-1.22) were more likely to be overweight/obese. Also, compared to women with zero parity, the odds of overweight/obesity was highest among women with three births in urban areas (aOR = 1.38, 95% CI = 1.24–1.54), but no significant difference was observed in rural areas. Furthermore, the odds of overweight/obesity was higher among women who watched television at least once a week compared to those who never watched television in both rural (aOR = 2.33, 95% CI = 2.21–2.45) and urban (aOR = 1.70, 95% CI = 1.60–1.81) areas. Similarly, the odds of overweight/obesity increased with increasing wealth index in both rural (aOR = 1.94, 95% CI = 1.78–2.12) and urban (aOR = 2.23, 95% CI = 1.96–2.53) areas, with those in the richest wealth index having the highest likelihood (Fig. 2). See Additional file 1: Table S2 for additional details on the results.

### Factors contributing to rural–urban disparities in overweight/obesity

Table 4 shows the results of the factors contributing to the rural–urban disparities in overweight/obesity among women in SSA. Overall, approximately 54% of the rural–urban disparities in overweight/obesity were attributable to the differences in the women’s characteristics/explanatory variables (Table 4). Therefore, if the disparities in women’s characteristics were levelled, more than half of the rural–urban disparities in overweight and obesity would be reduced. Among the women’s characteristics, frequency of watching television (29.03%), wealth index (26.59%), and level of education (9.40%) explained approximately 65% of the rural–urban differences in overweight/obesity (Table 4).

**Table 4:** Decomposition analysis of factors contributing to rural–urban disparities in overweight/obesity

Variables	Difference due to characteristics (E)		Difference due to coefficients (C)	
	Coefficient	Percent	Coefficient	Percent
% Total explained disparity	0.10163***	54.09	0.08624***	45.91
*R*	0.18787***			
Women’s age (years)				
15–19	− 0.00033***	− 0.18	0.00744***	3.96
20–24	− 0.00308***	− 1.64	0.00070	0.37
25–29	− 0.00040***	− 0.21	− 0.00068	− 0.36
30–34	0.00004***	0.02	− 0.00139*	− 0.74
35–39	− 0.00060***	− 0.32	− 0.00138*	− 0.74
40–44	− 0.00164***	− 0.87	− 0.00094	− 0.50
45–49	− 0.00250***	− 1.33	− 0.00032	− 0.17
Total		− 4.53		1.82
Marital status				
Never in union	− 0.00297***	− 1.58	− 0.00329*	− 1.75
Married	− 0.00238***	− 1.27	0.00251	1.34
Living with partner	− 0.00015**	− 0.08	− 0.00015	− 0.08
Widowed	0.00005*	0.02	0.00020	0.11
Divorced	0.00016*	0.08	0.00002	0.01
Separated	− 0.00007	− 0.04	0.00007	0.04
Total		− 2.87		− 0.33
Level of education				
No education	0.00936***	4.98	0.01941***	10.33
Primary	0.00040	0.21	0.00418**	2.22
Secondary	0.00550***	2.93	− 0.00522***	− 2.78
Higher	0.00241***	1.28	− 0.00084***	− 0.45
Total		9.40		9.32
Current working status				
No	0.00020*	0.11	− 0.00217**	− 1.15
Yes	0.00020*	0.11	0.00308**	1.64
Total		0.22		0.49
Parity				
Zero birth	− 0.00488***	− 2.60	− 0.00647***	− 3.45
One birth	− 0.00018	− 0.09	− 0.00110	− 0.58
Two births	0.00035***	0.19	0.00027	0.14
Three births	0.00008***	0.04	0.00131*	0.70
Four or more births	− 0.00208**	− 1.10	0.00812***	4.32
Total		− 3.56		1.13
Frequency of watching television				
Not at all	0.01160***	6.17	0.01281***	6.82
Less than once a week	0.00104***	0.55	− 0.00244***	− 1.30
At least once a week	0.04191***	22.31	− 0.00750***	− 3.99
Total		29.03		1.53
Household size				
Small	− 0.00009	− 0.05	− 0.00039	− 0.21
Medium	− 0.00026	− 0.14	0.00504***	2.68
Large	0.00001	0.00	− 0.00122**	− 0.65
Total		− 0.19		1.82
Wealth index				
Poorest	0.01854***	9.87	− 0.00462**	− 2.46
Poorer	0.00472***	2.51	− 0.00060	− 0.32
Middle	− 0.00079	− 0.42	0.00035	0.19
Richer	0.00444***	2.36	0.00258***	1.37
Richest	0.02304***	12.27	0.00013	0.07
Total		26.59		− 1.15
Constant			0.05872***	31.25

## Discussion

In this study, our findings revealed high prevalence of overweight/obesity among women in SSA (27.0%), which ranged from 7.4% in Ethiopia to 49.7% in Mauritania. We observed higher prevalence among women in urban areas than in rural areas in all the countries surveyed, except in South Africa where the prevalence was higher among those in rural areas compared to urban areas. Also, the likelihood of overweight/obesity was higher among women in urban areas relative to those in rural areas. Disparities in women’s characteristics such as educational level, wealth index, and frequency of watching television significantly influenced the observed rural–urban inequalities in overweight/obesity. If these disparities were levelled, more than half of the rural–urban inequalities in overweight/obesity among the women in SSA would be reduced.

Compared to previous studies [31, 32], we found high prevalence of overweight/obesity among women in SSA. In a previous study involving women from 32 countries in SSA, Neupane et al. [31] reported a pooled prevalence of 15.9% and 6.7% for overweight and obesity, respectively. Perhaps, our findings affirm the rising burden of overweight/obesity among women in many countries in SSA [33–35]. For example, an analysis of DHS data from 24 countries in SSA revealed that most of the countries surveyed experienced a significant rise in the prevalence of overweight and obesity between 1991 and 2014 [33]. Therefore, the current findings highlight the need for continuous monitoring and policy interventions to address the high prevalence of overweight/obesity among women and reduce the risk of escalating the burden of NCDs in SSA.

Moreover, we found higher prevalence of overweight/obesity among women in urban areas than in rural areas in all the countries surveyed, except in South Africa. Besides, the likelihood of overweight/obesity was higher among women in urban areas. The current findings support the findings of previous studies that reported higher burden of overweight/obesity among women in urban areas than those in rural areas in SSA [33, 36, 37]. One plausible reason for this finding is that women in urban areas often engage in less physical activities or more sedentary behaviours [37] and have increased access to highly refined foods and sugar-sweetened beverages [33]. This could result in energy imbalance as total calories expended tend to remain far lower than calories consumed and thus predisposing the women in urban areas to overweight/obesity [31, 38]. Therefore, increasing the level of physical activity among women in urban settings remains one of the best ways for reducing excessive energy accumulation and its associated risk for overweight/obesity [39]. Meanwhile, our findings in South Africa support a previous study [40] that reported higher prevalence of overweight/obesity among women in rural areas compared to those in urban areas in South Africa. Although the reasons for the higher prevalence of overweight/obesity among women in rural areas in South Africa remains unclear [40], the phenomenon have been partly attributed to the frequent population flow between rural and urban areas which is influencing the lifestyle of rural dwellers by increasing their intake of unhealthy diets and sedentary lifestyles [41]. Perhaps, further studies are needed to ascertain the patterns and factors predisposing women in rural areas to overweight/obesity in South Africa.

Several studies have attributed the growing trend of overweight and obesity in SSA to nutritional transition (from unrefined to highly refined foods, saturated fats, and sugar), and increased sedentary lifestyle, or reduced physical activity [18, 33, 34, 42]. Similar to the findings of previous studies in Mali [19], Ghana [20], Ethiopia [43], Nigeria [24], Zambia [21], and Uganda [22], we found that the odds of being overweight/obese increased with increasing frequency of watching television in both rural and urban areas, although higher odds were observed in rural areas. Available evidence suggests that increased frequency and prolonged television watching promotes overweight/obesity in several ways, including increasing sedentariness [44], displacement of leisure-time physical activity [45], altered sleep pattern [46], increased consumption of nutrient-poor diet (due to regular exposure to television advertisements), and unhealthy snacking while watching television [47]. Although television is a useful source of information particularly on women’s health matters [48], it is important to highlight its potential negative impact on health when used frequently. Perhaps, this could guide the women to limit their television screen time by focusing on watching only ‘essential’ television programmes.

Despite the higher prevalence of overweight/obesity among women in urban areas relative to those in rural areas, we observed that women in rural areas who watched television at least once a week were 2.3 times more likely to be overweight/obese while their counterparts in urban areas were 1.7 times more likely to be overweight/obese. The higher odds of overweight/obesity among women in rural women areas who watch television have been reported in previous studies in India [15] and in Myanmar [49]. It is argued that women in urban areas are exposed to myriads of overweight/obesity risk factors such as increased sedentary behaviours and eating energy-dense foods compared to those in rural areas, which offsets the significance of television viewing as a risk factor for overweight/obesity among women in urban areas [15, 49]. Perhaps, the current findings highlight the importance of television watching as a risk factor for overweight/obesity among women in rural areas in SSA. This could guide interventions targeted at reducing overweight/obesity among women in rural SSA. Nonetheless, we recommend further studies to provide deeper understanding of this phenomenon in SSA.

Further, we observed higher odds of overweight/obesity among women with higher wealth index as well as those with higher educational level in both rural and urban areas. The positive association between these socioeconomic indices and overweight/obesity have been reported in several previous studies in SSA [32, 35, 50]. In Nigeria for instance, Okoh [24] reported that women with tertiary education were about three and seven times more likely to be overweight and obese, respectively, compared to those with no formal education. Evidence suggests that women with higher socioeconomic status in SSA engage in more sedentary behaviour, and consumption of highly processed and energy-dense foods [32, 51], which increases their risk of overweight/obesity [35]. Perhaps, instituting public health interventions that promote physical activities such as regular exercise and avoidance of energy-dense foods could reduce the risk of overweight/obesity among wealthy and educated women in both urban and rural settings in SSA.

Also, we observed higher odds of overweight/obesity among women with advanced age relative to younger women in both rural and urban areas. Previous studies in SSA [19, 36] reported a positive association between women’s age and overweight/obesity. For instance, Mangemba and San Sebastian [52] found that women who were 40 years or older were more than five-fold more likely to be overweight/obese compared to those aged 15–19. This observation has been attributed to the relatively high accumulation of body fat and the marginal reduction in height often associated with increasing age [53]. Marital status and parity significantly predicted overweight/obesity among women, but in urban areas only. Previous studies identified high parity [24] and being married [20] as significant predictors of overweight and obesity among women in SSA. Plausibly, multiparous women as well as married women who reside in urban areas are more prone to sedentary lifestyles that increases their odds for overweight/obesity, and thus may benefit immensely from improved physical activity.

## Strengths and limitations

Our analysis was based on the most recent nationally representative datasets of 23 countries in SSA, which enhanced the robustness of the prevalence estimates. Additionally, the robust statistical analysis employed in the current study enhances the reliability of our findings and strengthens the observed inequalities and associations between the variables. Despite these strengths, our study has some limitations. First, our findings report only rural–urban inequalities and factors associated with overweight/obesity using a cross-sectional study design. Thus, we could not infer causality. Second, although the duration of television watching could contribute to the extent of television exposure, our analysis relied on only the frequency of television viewing, since data on duration were not available. Additionally, the DHS did not collect data on dietary habits and physical activity of the respondents. Hence, the results of this study should be interpreted with caution. Further, the large sample and the risk of false-positive error during the hypothesis testing is another limitation and call for caution in interpreting the results.

## Conclusion

The prevalence of overweight/obesity among women in SSA remains high and skewed towards women in urban areas. Increased frequency of watching television, high wealth index, and higher educational attainment were the major factors that contributed to the rural-urban disparities in overweight/obesity among women in SSA. Thus, interventions aimed at reducing overweight/obesity among women in SSA could be targeted at reducing the frequency of television watching as well as promoting physical activities among wealthy women and those with higher education, particularly in urban areas. Also, public health education and awareness creation could be intensified and highlighted to show the potential health implications of overweight/obesity.

### Supplementary Information

**Additional file 1: Table S1.** Distribution of overweight/obesity across the explanatory variables. **Table S2.** Details on factors associated with overweight/obesity among women in rural and urban sub-Saharan Africa

## Data Availability

The dataset is freely available to download at https://dhsprogram.com/data/available-datasets.cfm.

## Abbreviations

AIC: Akaike Information Criterion
aOR: Adjusted odds ratio
BMI: Body mass index
CIs: Confidence intervals
DHS: Demographic and Health Survey
NCD: Non-communicable disease
PSU: Primary sampling unit
SDG: Sustainable Development Goal
SSA: Sub-Saharan Africa
