# Supplementary material for: Residence-based inequalities in overweight/obesity in sub-Saharan Africa: a multivariate non-linear decomposition analysis
Source: Trop Med Health. 2024 Apr 7;52:29. doi: 10.1186/s41182-024-00593-5 (PMC10999097; doi:10.1186/s41182-024-00593-5)
Supplement: Supplementary file 1 — Additional file 1: Table S1. Distribution of overweight/obesity across the explanatory variables. Table S2. Details on factors associated with overweight/obesity among women in rural and urban sub-Saharan Africa [file 41182_2024_593_MOESM1_ESM.docx]

**Table S1: Distribution of overweight/obesity across the explanatory variables**

| **Variables** | **Pooled** | | **Rural** | | **Urban** | |
| --- | --- | --- | --- | --- | --- | --- |
|  | **% [CI]** | **P-value** | **% [CI]** | **P-value** | **% [CI]** | **P-value** |
| **Women’s age (years)** |  | <0.001 |  | <0.001 |  | <0.001 |
| 15-19 | 10.4 [10.0, 10.9] |  | 7.0 [6.6, 7.4] |  | 15.7 [14.8, 16.6] |  |
| 20-24 | 18.9 [18.3, 19.6] |  | 13.4 [12.7, 14.1] |  | 26.4 [25.2, 27.5] |  |
| 25-29 | 28.1 [27.3, 28.9] |  | 19.0 [18.2, 19.8] |  | 40.6 [39.3, 42.0] |  |
| 30-34 | 35.2 [34.3, 36.1] |  | 24.4 [23.5, 25.3] |  | 50.9 [49.4, 52.3] |  |
| 35-39 | 39.0 [38.0, 40.0] |  | 27.4 [26.5, 28.4] |  | 56.5 [54.9, 58.1] |  |
| 40-44 | 39.0 [37.9, 40.0] |  | 28.5 [27.4, 29.6] |  | 56.6 [54.8, 58.4] |  |
| 45-49 | 39.5 [38.3, 40.6] |  | 28.9 [27.7, 30.0] |  | 59.7 [57.7, 61.7] |  |
| **Level of education** |  | <0.001 |  | <0.001 |  | <0.001 |
| No education | 20.9 [20.3, 21.5] |  | 15.7 [15.1, 16.3] |  | 37.5 [36.1, 38.9] |  |
| Primary | 24.9 [24.3, 25.5] |  | 18.9 [18.3, 19.5] |  | 40.7 [39.4, 41.9] |  |
| Secondary | 30.8 [30.2, 31.5] |  | 22.6 [21.9, 23.3] |  | 36.7 [35.8, 37.5] |  |
| Higher | 45.5 [44.1, 46.9] |  | 42.9 [40.4, 45.4] |  | 46.2 [44.5, 47.9] |  |
| **Marital status** |  | <0.001 |  | <0.001 |  | <0.001 |
| Never in union | 17.8 [17.2, 18.3] |  | 11.4 [10.9, 12.0] |  | 24.0 [23.2, 24.9] |  |
| Married | 31.1 [30.5, 31.7] |  | 22.0 [21.5, 22.6] |  | 49.0 [48.0, 50.0] |  |
| Living with partner | 30.4 [29.3, 31.6] |  | 20.3 [19.2, 21.4] |  | 45.7 [43.7, 47.6] |  |
| Widowed | 32.6 [30.9, 34.4] |  | 23.3 [21.5, 25.3] |  | 49.7 [46.5, 52.8] |  |
| Divorced | 34.3 [32.3, 36.4] |  | 21.9 [19.9, 24.1] |  | 49.2 [46.1, 52.3] |  |
| Separated | 32.1 [30.5, 33.6] |  | 20.9 [19.3, 22.5] |  | 46.5 [43.9, 49.0] |  |
| **Current working status** |  | <0.001 |  | <0.001 |  | <0.001 |
| No | 23.8 [23.2, 24.4] |  | 17.1 [16.4, 17.7] |  | 32.4 [31.5, 33.3] |  |
| Yes | 29.3 [28.8, 29.8] |  | 20.4 [19.9, 20.9] |  | 44.1 [43.3, 44.9] |  |
| **Parity** |  | <0.001 |  | <0.001 |  | <0.001 |
| Zero birth | 16.1 [15.6, 16.6] |  | 10.5 [9.9, 11.0] |  | 22.2 [21.4, 23.1] |  |
| One birth | 26.6 [25.7, 27.4] |  | 16.2 [15.4, 17.0] |  | 38.4 [37.1, 39.8] |  |
| Two births | 32.8 [31.9,33.7] |  | 21.3 [20.4, 22.3] |  | 47.0 [45.6, 48.5] |  |
| Three births | 35.5 [34.5,36.5] |  | 24.2 [23.2, 25.2] |  | 51.9 [50.2, 53.5] |  |
| Four or more births | 31.7 [31.1, 32.4] |  | 23.5 [22.8, 24.1] |  | 52.5 [51.3, 53.7] |  |
| **Frequency of watching television** |  | <0.001 |  | <0.001 |  | <0.001 |
| Not at all | 17.8 [17.4, 18.3] |  | 15.1 [14.6, 15.5] |  | 29.8 [28.7, 30.9] |  |
| Less than once a week | 30.3 [29.2, 30.8] |  | 24.1 [23.2, 25.0] |  | 36.6 [35.3, 37.8] |  |
| At least once a week | 41.1 [40.4, 41.8] |  | 34.6 [33.6, 35.7] |  | 43.6 [42.8, 44.4] |  |
| **Household size** |  | <0.001 |  | <0.001 |  | <0.001 |
| Small | 29.0 [28.4, 29.5] |  | 20.2 [19.6, 20.8] |  | 40.9 [40.1, 41.8] |  |
| Medium | 25.6 [25.1, 26.2] |  | 18.4 [17.8, 18.9] |  | 37.9 [37.0, 38.9] |  |
| Large | 24.6 [23.6, 25.6] |  | 17.8 [16.8, 18.9] |  | 33.9 [32.3, 35.5] |  |
| **Wealth index** |  | <0.001 |  | <0.001 |  | <0.001 |
| Poorest | 13.6 [13.0, 14.2] |  | 12.6 [12.1, 13.2] |  | 23.0 [20.7, 25.6] |  |
| Poorer | 18.3 [17.7, 18.9] |  | 16.5 [15.9, 17.1] |  | 30.2 [28.1, 32.4] |  |
| Middle | 23.7 [23.0, 24.4] |  | 20.8 [20.0, 21.5] |  | 32.2 [30.7, 33.7] |  |
| Richer | 31.8 [31.1, 32.6] |  | 24.5 [23.5, 25.4] |  | 39.0 [37.9, 40.1] |  |
| Richest | 41.2 [40.4, 42.0] |  | 33.7 [32.1, 35.4] |  | 42.6 [41.8, 43.5] |  |
| **Overall prevalence** | 27.0 [26.6,27.5] |  | 19.1 [18.7-19.6] |  | 38.9 [38.2-39.6] |  |

**Table S2: Factors associated with overweight/obesity among women in rural and urban sub-Saharan Africa**

| **Variables** | **Rural** | **Urban** |
| --- | --- | --- |
|  | **aOR [95% CI]** | **aOR [95% CI]** |
| **Women’s age (years)** |  |  |
| 15-19 | 1.00 | 1.00 |
| 20-24 | 1.99^***^ [1.81, 2.19] | 1.65^***^ [1.51, 1.81] |
| 25-29 | 3.40^***^ [3.07, 3.77] | 2.80^***^ [2.54, 3.10] |
| 30-34 | 5.24^***^ [4.71, 5.84] | 4.12^***^ [3.70, 4.60] |
| 35-39 | 6.63^***^ [5.94, 7.39] | 5.29^***^ [4.71, 5.93] |
| 40-44 | 7.39^***^ [6.60, 8.27] | 5.51^***^ [4.87, 6.23] |
| 45-49 | 7.79^***^ [6.94, 8.74] | 6.57^***^ [5.78, 7.47] |
| **Marital status** |  |  |
| Never in union | 1.00 | 1.00 |
| Married | 1.11^*^ [1.02, 1.22] | 1.21^***^ [1.12, 1.32] |
| Living with partner | 1.07 [0.96, 1.18] | 1.24^***^ [1.12, 1.38] |
| Widowed | 0.95 [0.83, 1.09] | 1.00 [0.86, 1.17] |
| Divorced | 1.12 [0.97, 1.29] | 1.27^***^ [1.10, 1.47] |
| Separated | 0.95 [0.84, 1.08] | 1.09 [0.96, 1.25] |
| **Level of education** |  |  |
| No education | 1.00 | 1.00 |
| Primary | 1.48^***^ [1.41, 1.55] | 1.28^***^ [1.19, 1.38] |
| Secondary | 2.00^***^ [1.88, 2.12] | 1.39^***^ [1.30, 1.50] |
| Higher | 2.36^***^ [2.09, 2.67] | 1.40^***^ [1.28, 1.54] |
| **Current working status** |  |  |
| Not working | 1.00 | 1.00 |
| Working | 0.87^***^ [0.83, 0.91] | 0.95^*^ [0.90, 0.99] |
| **Parity** |  |  |
| Zero birth | 1.00 | 1.00 |
| One birth | 1.07 [0.97, 1.18] | 1.24^***^ [1.13, 1.36] |
| Two births | 1.12^*^ [1.00, 1.24] | 1.32^***^ [1.19, 1.46] |
| Three births | 1.11 [0.99, 1.24] | 1.39^***^ [1.24, 1.55] |
| Four or more births | 0.98 [0.88, 1.09] | 1.38^***^ [1.24, 1.54] |
| **Frequency of watching television** |  |  |
| Not at all | 1.00 | 1.00 |
| Less than once a week | 1.63^***^ [1.54, 1.72] | 1.29^***^ [1.19, 1.38] |
| At least once a week | 2.33^***^ [2.21, 2.45] | 1.70^***^ [1.60, 1.81] |
| **Household size** |  |  |
| Small | 1.00 | 1.00 |
| Medium | 0.95^*^ [0.91, 1.00] | 1.02 [0.96, 1.07] |
| Large | 1.01 [0.95, 1.08] | 0.95 [0.88, 1.02] |
| **Wealth index** |  |  |
| Poorest | 1.00 | 1.00 |
| Poorer | 1.26^***^ [1.19, 1.34] | 1.42^***^ [1.22, 1.66] |
| Middle | 1.47^***^ [1.39, 1.56] | 1.53^***^ [1.33, 1.75] |
| Richer | 1.63^***^ [1.53, 1.73] | 2.04^***^ [1.80, 2.32] |
| Richest | 1.94^***^ [1.78, 2.12] | 2.23^***^ [1.96, 2.53] |
| **Place of residence** |  |  |
| Rural |  |  |
| Urban |  |  |
| ***N*** | **108512** | **68817** |
| **pseudo *R*^2^** | **0.104** | **0.112** |

Exponentiated coefficients; 95% confidence intervals in brackets

^*^ *p* < 0.05, ^**^ *p* < 0.01, ^***^ *p* < 0.001
